# Multilevel Analysis of the Relationship between Ownership Structure and Technical Efficiency Frontier in the Spanish National Health System Hospitals

**DOI:** 10.3390/ijerph17165905

**Published:** 2020-08-14

**Authors:** Mª Isabel Ortega-Díaz, Ricardo Ocaña-Riola, Carmen Pérez-Romero, José Jesús Martín-Martín

**Affiliations:** 1 Departamento de Economía, Universidad de Jaén, Edificio D-3, Campus Las Lagunillas s/n, 23071 Jaén, Spain; iortega@ujaen.es; 2 Escuela Andaluza de Salud Pública, Cuesta del Observatorio 4, Campus Universitario de Cartuja, 18011 Granada, Spain; ricardo.ocana.easp@juntadeandalucia.es; 3 Instituto de Investigación Biosanitaria ibs.GRANADA, Doctor Azpitarte 4, 18012 Granada, Spain; jmartin@ugr.es; 4 Departamento de Economía Aplicada, Universidad de Granada, Facultad de Ciencias Económicas y Empresariales, Campus Universitario de Cartuja s/n, 18071 Granada, Spain

**Keywords:** efficiency, data envelopment analysis, binomial analysis, public hospitals, private hospitals, public-private partnership, economic crisis, COVID-19

## Abstract

*Objective:* To evaluate the relationship between the ownership structure of hospitals and the possibility of their being positioned on the frontier of technical efficiency in the economic crisis period 2010–2012, adjusting for hospital variables and regional characteristics in the areas where the Spanish National Health System (SNHS) hospitals are located. *Methods*: 230 National Health System hospitals were studied over the two-year period 2010–2012 according to their ownership structure—public hospitals, private hospitals and public–private partnership (PPP)—data envelopment analysis orientated to inputs was used to measure the overall technical efficiency, pure efficiency and efficiency of scale. A generalised linear mixed model (GLMM) with binomial distribution and logit link function was used to analyse the hospital and regional variables associated with positioning on the frontier. *Results:* There are substantial differences between the average pure technical efficiency of public, private and PPP hospitals, as well as a greater number of PPP models being positioned on the efficiency frontier (91.67% in 2012). The odds of being positioned on the frontier are 41.7 times higher in PPP models than in public hospitals. The average annual household income per region is related to the greater odds of hospitals being positioned on the frontier of efficiency. *Conclusions:* During the most acute period of recession in the Spanish economy, PPP formulas favoured hospital efficiency, by increasing the odds of being positioned on the frontier of efficiency when compared to private and public hospitals. The position on the frontier of efficiency of a hospital is related to the wealth of its region.

## 1. Introduction

Some of the main challenges currently faced by health systems in developed countries are the coverage of the health care needs of an increasingly ageing population, with a multitude of chronic diseases and the growing costs of technological innovation. The recent worldwide financial crisis, and the consequent budget restrictions on the provision of state services, has encouraged public health systems to develop various strategies to make management more flexible, in order to guarantee financial sustainability [1]. The introduction of public-private partnership (PPP) formulas stands out among these new measures. The aim of these formulas is to incorporate management tools similar to those used by private companies into the public health sector, while maintaining public ownership of the service.

This study focuses on the most acute period of recession in the Spanish economy, 2010–2012, when public healthcare spending per capita fell from 1510 euros in 2009 to 1357 euros in 2012 [2], in order to analyse the degree of resilience of different hospital ownership structures (public and private hospitals and PPP models), and position them on the efficiency frontier in adverse economic circumstances with severe financial restrictions.

PPP is defined as a long-term contract between a private agent and a government agency to provide an asset or a public service, in which the private agent assumes risk and management responsibility [3,4]. These formulas have experienced enormous growth in recent years as contractual systems for public-private collaboration, with a view to improving efficiency and guaranteeing sustainability in various fields of activity (energy companies, knowledge intensive business services industry, etc.) [5,6], as well as hospitals [7,8].

The lessons learned from the experience with PPPs in public health systems have allowed for the implementation of elements characteristic of the free market, such as separation of purchasing and service provision functions and the transfer of risk from the financier to the provider [9]. After a decade of experience, the contribution made by PPP formulas in the healthcare sector [10,11] regarding budgetary adjustment, the improvement of public resource use and efficiency results [12] has led to a new debate.

In Europe, there has been extensive experience of outsourcing public health services through PPP models, especially in the hospital sector. The United Kingdom’s National Health Service pioneered PPP use in the early 1990s; this example has been followed by most European countries [9]. Outsourcing has been deployed both in the supply of logistical or structural services, as well as in the provision of specific clinical procedures. Complete outsourcing initiatives for hospital care have also been developed [13,14]. Traditionally, in the Spanish National Health System (SNHS), outsourcing health services has been carried out to a lesser extent, with the exception of Catalonia, where the public health network is traditionally made up of public and private providers. However, in recent decades, several PPP formulas have been rolled out intensively. This has tested indirect hospital care management [15]. There are two categories of the aforementioned PPP concession model [9]: The Health Administrative Concession and the concession of work or Private Finance Initiative (PFI). The difference between the two models lies in the scope of the contract. Thus, while the health administrative concession category involves the complete outsourcing of hospital management (clinical and non-clinical services), in PFI models, the private company only takes on the management of logistical or structural services at health centres, the public sector retains its management of the hospital’s clinical services.

Analysis about the effect of public or private ownership of hospitals on efficiency has traditionally been built on the efficiency frontier concept. Frontier models base their methodological strategy explicitly on the construction of an efficiency frontier, based on the hospitals considered to be the best within a set of study hospitals [16]. The two main methods used to compare the efficiency of public and private hospitals are the stochastic frontier and, more widely used in healthcare, data envelopment analysis (DEA). The DEA approach offers the possibility of constructing a nonparametric frontier of efficiency, which considers the multiple inputs and outputs that are characteristic of hospital production [17,18,19,20,21,22].

Once the technical frontier of efficiency has been obtained, the identification of variables related to the degree of efficiency of hospitals, known as “second stage analysis”, is frequently performed using bivariate analysis or multivariate regression models [23,24,25]. Environmental or contextual factors, such as geographical location or population [26,27], competition in the sector [28], hospital characteristics [29] and ownership [30], among others, tend to be established as independent variables or control variables in the regression models used in second stage analyses [31].

This paper builds on the existing literature and our own previous research [32,33] in three relevant aspects. Firstly, as opposed to the previous literature, this analysis specifically distinguishes between public, private and PPP hospital models. Secondly, it studies which of the three models provides the greatest odds of being positioned on the efficiency frontier. This analysis differs from the conventional analysis, which focuses on establishing the relationship between type of ownership and level of efficiency. Finally, due to the decentralised nature of the SNHS, which has transferred health management responsibilities to regional bodies, a regional element has been incorporated, to investigate whether population ageing, the dimension of the private health sector, wealth and public services spending are related to the possibility of hospitals being positioned on the efficiency frontier or not. Methodologically, this involves modifying the usual approach to the second stage, and replacing the linear multivariate analysis with a generalised linear mixed model (GLMM), with binomial distribution and logit link function and the hierarchical structure of the data, so that it addresses the research objective of being positioned on the efficiency frontier or not.

Formulated in terms of objectives, there are two. Firstly, to consider the three different structures of hospital management and estimate the odds of each structure being on the efficiency frontier and, secondly, to determine whether the demographic characteristics, public social spending and wealth of the hospital’s region are related to being placed on the frontier of efficiency.

Recent studies in European countries have evaluated the technical efficiency of public and private hospitals using DEA, traditionally differentiating between three types of ownership: public hospitals, private for-profit hospitals and private non-profit hospitals. Examples of this work have been carried out in Italy [34,35], Germany [36,37,38,39], Austria [21,40], Portugal [41] and Spain [32,33]. The results point to an ambiguous and contradictory relationship between hospital ownership and technical efficiency, depending on the incentive structure provided by the regulatory framework to which they are subject [9,10,11]. As Shen et al. [42] suggest, the differences can be explained by the diversity of the national incentive structures of the respective hospital financing systems, or, as Pérez-Romero et al. [33] posit in Spain, it is the hospital regulatory and management frameworks, rather than public or private ownership, which explain the differences in technical efficiency.

Unlike the relative abundance of studies using efficiency frontier models on public and private hospitals, the available evidence on the efficiency of PPP models compared to other hospital management structures is based primarily on case studies using partial indicators [13,43,44,45]. There is no agreement on whether PPP models are more or less efficient compared to more conventional models [14,46,47,48,49,50,51,52].

There are few prior studies which compare the structure of public, private, and mixed ownership (PPP models), as has been done in this research. In Spain, only two studies using regional frontier methods (DEA) with regional scope have been identified [53,54]. Their results indicate that PPP models obtain high levels efficiency on indices, often located on the frontier of technical efficiency, although in terms of change in productivity, PPPs are not always better than traditional public hospitals. However, the small number of units analysed limits the robustness of the results obtained.

The use of multi-level or hierarchical models to identify variables related to the technical efficiency of hospitals obtained with DEAs is scarce. Min et al. [55,56] use a cross-sectional multilevel analysis to identify the organisational factors which contribute to the level technical efficiency of nursing care in intensive care units in the United States, differentiating between nursing units, hospitals, and counties. An interesting finding in these studies is that the magnitude of the technical efficiency differential in nursing care varies markedly by county. While the results reveal that the environmental factors analysed (Medicare Advantage penetration and hospital competition in the market) do not generally favour a higher level of efficiency in intensive care units, they do, however, favour efficiency in the case of units with a certain level of efficiency. This would justify different management strategies and health policy measures within a region, based on the level of efficiency at each centre. Zhang et al. [57] apply a multi-level longitudinal approach to assess changes in and the determinants of efficiency during the reform of the Chinese healthcare system. The five independent variables considered (year, region, gross domestic product per capita, population density, and quantity of primary health workers) were statistically significant in determining the efficiency scores obtained with the DEA. In two recent studies carried out by our research team [32,33], the efficiency of all general hospitals in the SNHS was analysed by identifying the hospital and regional variables related to technical efficiency, using multilevel linear models. The results point to a significant direct linear relationship between technical efficiency, annual per capita income and spending on fundamental public services, and to an indirect relationship between the aging index and per capita public health spending. Having a legal personality is also seen to notably favour the efficiency of a hospital. The framework of hospital regulation and management is also found to be more relevant than whether it is publicly or private owned.

There is little prior research that identifies the factors related to being positioned on the frontier of technical efficiency or not [58,59,60]. All of it primarily uses the data envelopment analysis methodology to classify hospitals as efficient and inefficient. In second stage analysis, it used the previous categorical dependent variable in a logistic regression model to identify different hospital or environmental characteristics related to whether or not the hospital belongs on the best practices’ frontier. In the case of Lee et al. [58], a set of variables measuring organisational and environmental factors (specialisation, size, ownership, teaching status, organisational type, location and population) related to 106 urban hospitals located in Seoul are used as explanatory variables. Wilson et al. [59] use a set of six financial monitors as explanatory variables to achieve the objective of identifying the financial performance measures associated with the efficiency of rural hospitals in the United States. Finally, Kang et al. [60] focus their analysis on the emergency departments of 449 U.S. hospitals to determine best practices. In the second stage analysis, they analyse the particular characteristics of these departments, which contribute to their being considered efficient.

To the best of our knowledge, this is the first study to analyse the factors related to being positioned on the frontier of efficiency or not, while simultaneously investigating the structure of ownership (breaking it down into public, private and PPP hospitals), as well as factoring in the regional characteristics of where the hospitals are located, using a mixed model of random effects with binomial distribution and extra-binomial parameter to do so.

## 2. Materials and Methods

### 2.1. Scope of the Study

General hospitals owned publicly, privately or made up of public-private partnership models which were part of the SNHS during the period 2010–2012, when the Spanish economy had an acute period of recession [61,62,63] (Table A1 in Appendix A).

### 2.2. Units of Analysis

These include all general hospitals in the 17 regions which make up Spain which met the following inclusion criteria: having more than 50 installed beds, recorded activity in the emergency department, information available on all inputs and outputs considered (*n* = 230 hospitals) (Table 1).

### 2.3. Variables

The inputs used are the beds installed (proxy variable of the capital most used in the literature [20]), hired personnel (differentiating between medical personnel, other health personnel and non-health personnel) and spending on purchases and external services acquired. The outputs used are case-adjusted discharges (hospital discharges weighted by the case-mix index), outpatient consultations, outpatient emergencies and major outpatient surgical procedures (Table 2).

In each hospital, high-tech equipment, training of specialists and the ownership of the health centre are also considered. Each Spanish region includes a population variable (aging index), a wealth variable (average annual income per household), a regional public social spending variable (spending on fundamental public services per capita) [24] and a private health sector dimension variable (private hospital beds per 1000 inhabitants).

### 2.4. Sources of Information

The main sources of information used are the Statistics on Specialised Healthcare Centres (SIAE), the Basic Minimum Hospitalisation Data Collective (CMBD-H) and the National Hospital Directory, all published by the Spanish Ministry of Health, Consumption and Social Welfare.

### 2.5. Data Analysis

The data analysis consists of two phases [32,33,64]:

(1) Analysis of static efficiency by means of DEAs, to find out the overall technical efficiency (OTE), pure technical efficiency (PTE) and scale efficiency (SE) of each hospital.
(2) Second stage analysis is to identify factors related to the positioning of hospitals on the technical efficiency frontier, using a generalised linear mixed model (GLMM) with binomial distribution and logit link function [65].

The DEA is a deterministic non-parametric frontier method which compares the technical efficiency of organisations (DMU, decision making units) which operate in a similar environment, are homogeneous and present multidimensional inputs and outputs. With DEA, the technical efficiency of each hospital in the SNHS is obtained through the resolution of a mathematical programming problem, where the outputs and inputs of each hospital are weighted to maximise the quotient between outputs and inputs. These weights then determine the best possible placement of the evaluated centre in respect to the others of the same weight. The OTE offered by the DEA (obtained with the resolution of a CCR model [66]) is composed by the PTE (resolution of a BCC model [67] and by the SE (quotient of the OTE and PTE scores). The former measures the optimal use of productive factors, and the latter establishes the degree to which the organisation produces, in optimal dimension, according to its size, assuming variable returns to scale. The frontier is established by the healthcare units considered to be efficient, because they achieve an index of 1 and any linear combination thereof.

In this study, input orientated DEA is carried out. That the health policies promoted in Spain in the years analysed prioritised the control of costs is assumed [20,64,68]. This makes it possible to explore which units are capable of maintaining their level of production, using, in relative terms, fewer resources. The analytical formulation of the DEA with variable yields of scale and oriented to input in its enveloping version is [69]:

Min θ(1)

Subject to

(2) $$\overset{n}{\underset{j=1}{\sum }}\lambda_{j}\chi_{ij\;}\leq \;\theta \chi_{i0}\;,\;\;i=1,\;\ldots ,\;m$$

(3) $$\overset{n}{\underset{j=1}{\sum }}\lambda_{j}y_{rj\;}\geq \;y_{r0}\;,\;\;r=1,\;\ldots ,\;s$$

(4) $$\overset{n}{\underset{j=1}{\sum }}\lambda_{j}{}_{\;}=1$$

(5) $$\lambda_{j}\geq 0\;,\;\;j=1,\;\ldots ,\;n$$

This optimisation programme is formed by a vector of *n* hospitals constituted of m inputs and s outputs, so that χ*ij* is the amount of input *i* consumed by the DMU *j* (existing *n* DMUs), Y*rj* is the amount of output r produced by the DMU *j*, *θ* is the proportion in which inputs can be reduced and λ*j* the intensity of the DMU *j* in the construction of the reference DMU. The analytical formulation of the DEA with constant scale yields is obtained by eliminating the restriction:

(6) $$\overset{n}{\underset{j=1}{\sum }}\lambda_{j}=1\;,\;j=1,\;\ldots ,\;n$$

Inputs and outputs have been selected based on the literature, including, as widely as possible, a set of resources and hospital production [70,71] and its isotonic character (Table 2). Additionally, the rule is proposed by Banker et al. [72], where the number of DMU ≥ max {inputs × outputs, 3 × (inputs + outputs)}; (230 ≥ max {20, 27}) is upheld. The robustness of the base model has been contrasted by defining five alternative models, checking the consistency of the results using the Spearman correlation coefficient.

A generalised linear mixed model (GLMM), with binomial distribution allowing for over dispersion throughout an extra-binomial parameter [65], was used to study the variables related to the hospital being positioned on the frontier of technical efficiency. This modelling considers the longitudinal design of the study and the hierarchical structure of the information, where the annual measurements (level 1) are grouped by hospitals (level 2) and then, in turn, by regions (level 3). Thus, the variable belonging to the technical efficiency frontier, *f_ijk_* (with a value of 1 if the hospital is positioned on the technical frontier of efficiency and 0 otherwise), is modelled as follows:

(7) $$f_{ijk}∼Binomial\left(1,p_{ijk}\right)$$

(8) $$var\left(f_{ijk}|\;p_{ijk}\right)=\theta p_{ijk}\left(1-p_{ijk}\right)$$

(9) $$logit\left(p_{ijk}\right)=\beta_{0jk}+\beta_{1j}x_{1ijk}+\overset{l^{\left(1\right)}}{\underset{r=1}{\sum }}\beta_{r}^{\left(1\right)}x_{rijk}^{\left(1\right)}+\overset{l^{\left(2\right)}}{\underset{r=1}{\sum }}\beta_{r}^{\left(2\right)}x_{rjk}^{\left(2\right)}+\overset{l^{\left(3\right)}}{\underset{r=1}{\sum }}\beta_{r}^{\left(3\right)}x_{rk}^{\left(3\right)}$$

(10) $$\beta_{0jk}=\beta_{0}+u_{0jk}+v_{0k}$$

(11) $$\beta_{1j}=\beta_{1}+u_{1jk}$$

where $f_{ijk}$ is the value of the dependent variable for the temporal measurement *i* = 1, 2, 3 of the hospital *j* = 1, ..., 230 in the region *k* = 1, ..., 17. The distribution of this variable is binomial with probability $p_{ijk}\;$and variance proportional to an extra-binomial parameter *θ*. This parameter can be set equal to 1 when there is a perfect fit to the binomial distribution, or it can be estimated on the basis of the data to take into account a possible extra-binomial variation. The probability of being positioned on the technical efficiency frontier, $p_{ijk}$, transformed by the logit function, is modelled through time centred on the first year, represented by $x_{1ijk}$, and a linear combination of the independent variables $x_{rijk}^{\left(1\right)}$, $x_{rjk}^{\left(2\right)}$, $x_{rk}^{\left(3\right)}$ representing, respectively, the variables of level 1(*r* = 1, …, l(1)), level 2 (*r* = 1, …, l(2)) and level 3 (*r* = 1, …, l(3)) centred on the mean. The independent term $\beta_{0jk}\;$and the dependent $\beta_{1j}\;$are considered random effects, which allows for the estimation of different time trends in each of the different hospitals. The errors of these random effects, represented respectively by $u_{0jk}$ and, $u_{1jk}$ follow a normal distribution with mean 0, variances $\sigma_{u_{0}}^{2}\;$and $\sigma_{u_{1}}^{2}\;$and covariance.$\sigma_{u_{01}}$ The error $v_{0k}$ is distributed according to a normal of mean 0 and variance$\;\sigma_{v_{0}}^{2}$. The coefficients of the rest of the independent variables, represented respectively by $\beta_{r}^{\left(1\right)}$ (*r* = 1, …, l(1)), $\beta_{r}^{\left(2\right)}$(*r* = 1, …, l(2)) and $\beta_{r}^{\left(3\right)}$ (*r* = 1, …, l(3)), are fixed effects.

The estimation of the model parameters was performed using the iterative generalised least squares (IGLS) method, with approximation of marginal quasi-verisimilitude through first-order Taylor series [65]. Once the model is estimated, the exponential coefficient of each independent variable represents the odds ratio (OR), adjusted by the remainder of the variables.

Both exogenous hospital variables and regional variables potentially related to the positioning of hospitals on the frontier of technical efficiency (Table 3) have been included as independent variables in this study. Likewise, in the analysis model, SNHS general hospitals have been classified according to their ownership, differentiating between public and private hospitals and PPP formulas.

## 3. Results

Table 4 shows the technical efficiency indices of the SNHS hospitals according to their ownership. An average has been obtained from the efficiency achieved by each of the general hospitals classified in each comparison group. The number of hospitals included in each group does not influence the technical efficiency indices obtained, given that the DEA compares each hospital with the set of hospitals in the SNHS. The robustness of the final analysis model has been contrasted by defining five alternative models (As shown in Table A2 in Appendix A), verifying the consistency of the results by means of the Spearman correlation coefficient.

There is a notable difference between the technical efficiency of public hospitals and that of private hospitals and PPPs over the three years, which the study analyses. In 2012, public hospitals had a lower average OTE index (0.706) than private hospitals (0.941) and PPP models (0.966). The percentage of efficient public hospitals (4.04%) is much lower than the percentage of private hospitals (60%) and PPP (58.33%) that are located on the frontier of maximum efficiency. When adjusting for the scale of production, the differences in technical efficiency decrease, although they are still present. In 2012, the average PTE of public hospitals stood at 0.809, of which 16.16% were efficient (PTE = 1). In addition, PPP formulas maintained a high level of efficiency (0.98), with 91.67% on the efficiency frontier.

Table 5 shows the results of the second stage analysis. The defined multilevel model showed that, between 2010 and 2012, the odds of being positioned on the technical efficiency frontier decreased on average 15.2% annually, after adjusting for the effect of the other variables. This change showed variations between hospitals, as shown by the variance in the time coefficient (0.46).

The odds of being positioned on the frontier of technical efficiency was 11.9 times greater in private hospitals than in public hospitals. This was 41.7 times greater in PPP hospitals than in public hospitals.

In terms of regional variables, the odds of being placed on the efficiency frontier increased 1.24 times for each 1000 Euro increase in average annual income per household. The rest of the regional variables considered, such as the aging index, spending on fundamental public services per capita and private beds per 1000 inhabitants, were not statistically significant.

## 4. Discussion

This paper is the first to study the relationship between the public, private and PPP formulas in SNHS hospitals and their position on the efficiency frontier, through a non-parametric frontier model (DEA), adjusting for regional and hospital variables. The paper also includes a second stage analysis using a mixed model of random effects with binomial distribution and extra-binomial parameter. The specification of the second stage analysis makes it possible to consider, both robustly and simultaneously, the regional and hospital variables associated with being positioned on the frontier.

In addition, the effect of PPP formulas on the technical efficiency of hospitals compared to alternative direct and indirect management models was investigated. There are very few previous studies that compare the structure of public, private and mixed ownership (PPP models) [53,54], as has been done in this research. Our results indicate that the configuration of PPP models favours efficiency. In Spain, PPPs are more efficient than traditional public hospitals. This highlights the positive effect of these formulas compared to direct hospital management, a question which previous work, carried out using different methodologies, doubted [13,43,44]. However, the average technical efficiency of PPPs is similar to that of private (for-profit and non-profit) hospitals. When compared, neither ownership structure clearly demonstrates higher levels of efficiency.

When analysing the hospital and environmental variables related to the positioning of hospitals on the frontier of efficiency, our work incorporates two developments in respect to the previous literature. Firstly, the results reveal that, between 2010 and 2012, the odds that SNHS hospitals were positioned on the technical efficiency frontier was 41.7 times higher in PPPs than in traditional public hospitals. Although there are previous studies that carry out second stage analyses to determine the impact that the type of ownership has on the efficiency of hospitals, none of them have determined the likelihood of PPPs achieving maximum technical efficiency compared to other models of hospital management [33,36,40,53,54,73,74,75,76]. On the other hand, previous studies that use a logit analysis and incorporate the type of ownership among the explanatory variables [58,77,78], do not reach a conclusive result about its influence, nor do they analyse the PPP formula in a differentiated manner. For example, Lee et al. [58], who use a logit analysis to determine whether the type of ownership is a significant variable when explaining the odds of a hospital being located on the efficiency frontier, only distinguishes between public and private hospitals. The study was based on information from a sample of hospitals which were located exclusively in Seoul, and were not randomly selected and located exclusively in Seoul. Our study, however, includes the set of hospitals which make up the entire SNHS, and are, therefore, subject to a similar institutional regulatory framework.

Knowing which type of ownership and management structure has the best chance of being positioned on the frontier of efficiency is very relevant in terms of health planning, as it would allow political decision-makers to guide the structure of public hospital supply design.

The period studied 2010–2012, the years when the Spanish economy was in a profound recession after the financial crisis of 2008, could make the results of this study particularly relevant in the near future, as a consequence of the profound financial crisis created by the COVID-19 pandemic. The International Monetary Fund (IMF) forecasts negative growth of 4.9% worldwide for 2020, 8% for the Eurozone and 12.8% for Spain [79]. Although projections for 2021 suggest an anaemic and uncertain recovery, the enormous fiscal deficit and increased public debt in many countries, and in particular, in Spain, will heavily condition the availability of the public resources which finance hospitals. This gloomy scenario makes the search for efficient hospital management structures more pressing in the context of recessions, such as those discussed in this paper.

However, the results obtained in this work, showing the greater technical efficiency of PPP formulas, are contingent on the institutional and regulatory framework of the SNHS. Spanish public hospitals mostly lack their own legal personality; they are decentralised cost centres with cost reimbursement budgetary systems. The labour relations model is one of civil service with remuneration systems dissociated from the results obtained by the hospital [80,81]. The replication of the methodological approach adopted in this study in other health systems could offer different results derived from different regulatory frameworks, to which the different types of hospitals analysed are subject.

Secondly, this study has taken advantage of the opportunity provided by the highly decentralised structure of the SNHS to analyse the influence that regional differences have on the positioning of hospitals on the frontier of technical efficiency. The results obtained reveal that the odds of Spanish hospitals being placed on the efficiency frontier increased 1.24 times, with each increase in the average annual income of 1000 euros per registered household in the region in which the hospital is located. In previous studies, the same regional variables that could be related to location on the frontier have not been analysed. Other studies focus on the rural or the location of the hospital is rural or urban or they reference population size, along with other hospital characteristics [58,59,60]. Our results suggest that only the per capita income level of a region is related to being positioned on the frontier, i.e., the greater the prosperity of a region, the greater its odds of being positioned on the frontier of efficiency, regardless of the specific hospital characteristics. These results, to the extent that they are confirmed by subsequent analysis, are relevant in the evaluation of hospital efficiency, given that efficiency may be related to regional ecological variables beyond the control not only of hospital directors, but also of regional health authorities. This aspect is especially important in decentralized health systems such as the SNHS. The result is contingent on the institutional structure of Spain’s health system and the lack of similar studies preventing comparisons.

The analysis carried out has several limitations. Firstly, the specifics of the methodology used derive from its deterministic character [66,82]. In order to reduce this problem, five alternative models have been contrasted. Secondly, the period of time analysed is short, and must be extended, both to observe the dynamic evolution, and to obtain, with longitudinal models, more robust estimates of the explanatory variables of the positioning of hospitals on the frontier of efficiency. Thirdly, there may be specification and omitted variable problems that could modify the impact of the analysed variables on technical efficiency. Last but not least, no variables have been included that specifically incorporate the dimension of quality in the complete measurement of hospital outputs used [83], as well as the analysis of the trade-off potential that could exist between efficiency and quality in the provision of services by hospitals with different ownership structure or management formula [84]. However, this is an omnipresent problem in all efficiency analyses (deterministic or stochastic) frontier and non-frontier, derived from the absence of widespread measurements of hospital activity quality.

The results obtained in this work suggest the development of four future lines of research, with which we could broaden our knowledge of the relationship between ownership structure and the technical efficiency frontier in Spanish National Health System hospitals. One of them would be the incorporation of the quality dimension in the provision of health services, although this would require overcoming existing methodological difficulties. Secondly, it would be of great interest to determine whether the relatively more efficient behaviour of PPP formulas is present at times of economic expansion. To this end, output-orientated DEA models, which would explore the potential improvements in levels of hospital production in the different management models, should be developed. Another relevant aspect would be the expansion of one of the main findings of this study, the existence of regional variables which relate to the likelihood of being positioned on the technical efficiency frontier or not, in order to verify if there are other ecological variables which influence the position of hospitals on the frontier. Finally, the geographical scope could be reduced to the provincial level into which the different regions of the Spain are divided using the generalised linear mixed model (GLMM), with binomial distribution and logit link function for analysis. This would allow analysis of whether there are different characteristics (demographic, economic, public spending on transversal policies, etc.) which influence the likelihood of hospitals being placed on the technical efficiency frontier or not in different provinces.

## 5. Conclusions

The proposed methodological approach integrates in an analytical framework, DEA with a second stage analysis by means of a mixed model of random effects with binomial distribution and extra-binomial parameter, which allows a simultaneous analysis of which variables at hospital and regional level are related to being positioned on the frontier of technical efficiency or not.

PPP models present the greater odds of positioning themselves on the frontier of technical efficiency than private and public hospitals in the SNHS, the latter presenting as having the least odds. These results are probably idiosyncratic of the SNHS and its institutional and organisational structure, as well as the economic crisis period (2010–2012) analysed, and need to be replicated in other health systems to assess the degree of generality. However, the consistent findings of this study suggest that PPP formulas have a better response in recession periods than other hospital management models.

Different types of hospitals being positioned on the efficiency frontier or not is linked to their regional characteristics, average annual household income in particular. This implies that hospital efficiency is related to some regional characteristics beyond the control of those responsible for hospitals.

## Figures and Tables

**Table 1:** Spanish National Health System general hospitals by ownership, structure and region.

Region	Public Hospitals	Private Hospitals	Public-Private Partnership	Total
Andalusia	31			31
Aragon	10			10
Principality of Asturias	8	1		9
Foral Community of Navarre	2			2
Canary Islands	7			7
Cantabria	3			3
Castile and Leon	14			14
Castillla—La Mancha	13			13
Catalonia	28	17		45
Valencian Community	21		5	26
Extremadura	6			6
Galicia	14	1		15
Balearic Islands	5			5
La Rioja	2			2
Community of Madrid	17	1	7	25
Basque Country	9			9
Region of Murcia	8			8
National Health Service	198	20	12	230

Source: Prepared by the authors, based on the annual information provided by the National Hospital Directory by the Spanish Ministry of Health, Consumption and Social Welfare.

**Table 2:** Input-output variables of the Spanish National Health Service general hospitals. 2010–2012.

	2010				2011				2012			
	Mean	Standard Deviation	Maximum	Minimum	Mean	Standard Deviation	Maximum	Minimum	Mean	Standard Deviation	Maximum	Minimum
**Inputs**												
Installed Beds ^a^	412.10	345.01	1673.00	56.00	411.17	341.94	1671.00	56.00	405.04	333.14	1671.00	56.00
Faculty Personnel ^b^	290.53	235.12	1139.00	23.00	291.19	241.01	1117.00	22.00	288.00	232.08	1082.00	21.50
Other Healthcare Personnel ^b^	1424.55	1314.40	6754.50	131.00	1387.15	1293.53	6847.50	129.50	1339.12	1239.48	6296.00	128.00
Non-Healthcare Personnel ^b^	435.76	442.27	2664.00	18.50	429.30	440.57	2665.00	17.00	410.46	405.56	2290.00	17.00
Purchases and external services ^c^	51,916.23	49,689.19	272,896.68	4812.35	54,256.62	53,112.82	259,344.72	4425.63	55,120.09	53,217.49	257,282.47	4415.69
**Outputs**												
Total Discharges Adjusted Case by Case ^d^	15,542.23	13,774.37	60,998.41	1840.80	15,554.50	13,666.87	60,259.96	1850.00	15,537.44	13,656.19	59,640.14	2074.42
Outpatient Consultations ^e^	290,880.27	223,693.08	1,135,574.00	49,141.00	295,460.60	222,401.22	1,047,253.00	43,363.00	291,776.89	217,721.86	1,049,911.00	44,724.00
Non-Admitted Emergencies ^f^	71,438.40	50,154.27	313,866.00	3000.00	72,023.20	50,129.06	309,757.00	3423.00	68,477.03	47,501.09	288,293.00	51.00
Major Surgical Procedures ^g^	3829.22	3117.22	18,785.00	0.00	3962.86	3218.28	20,124.00	0.00	4114.90	3310.22	2954.00	0.00

^a^ Annual average of the installed allocation, regardless of whether or not they have actually been in operation throughout the year. ^b^ Includes number of full-time professionals and number of part-time professionals (calculating part-time work at 50%). ^c^ Thousands of Euros. ^d^ Hospital discharges adjusted by applying the average Spanish weight (also known as case-mix index or casuistry index). The average weight is defined as the weighted average of the weights of the Diagnosis-Related Groups of all patients in a given unit, group or provider. ^e^ Integrates both outpatient consultations attended in the hospital itself and in its peripheral specialty centres. ^f^ Patients treated in hospital emergency departments who do not require admission or who have voluntarily discharged the patient, transferred to another health centre, or died. ^g^ Subsidiary processes of surgery performed under general anaesthesia, local anaesthesia or sedation that do not require hospital admission. Source: Prepared by the authors, based on Statistics on Specialised Healthcare Centres (SIAE) and the Basic Minimum Hospitalisation Data Collective (CMBD-H) of the Spanish Ministry of Health, Consumption and Social Welfare.

**Table 3:** Regional and hospital variables in the second stage analysis in the evaluation of technical efficiency of Spanish National Health System general hospitals. 2010–2012.

Quantitative Variables	Mean			Percentage Variation 2010–2012	Standard Deviation		
	2010	2011	2012		2010	2011	2012
**Exogenous Hospital Variables**							
Units of high-tech equipment per 100 faculty members	2.09	2.11	2.09	−0.01%	3.22	2.84	2.82
Resident Physicians per 100 faculty members	18.53	19.46	20.71	11.74%	17.37	17.14	17.49
Percentage of discharges not financed by the SNHS ^b^	4.95	4.73	3.82	−22.87%	13.10	13.30	9.17
**Regional Variables**							
Per Capita Spending on fundamental public spending ^c^ (Hundreds of €)	2736.15	2657.19	2477.92	−9.44%	286.94	277.59	301.4
Private Beds per 1000 inhabitants	0.55	0.54	0.53	−3.63%	0.35	0.35	0.33
Ageing Index	119.30	120.28	121.17	1.57%	36.31	36.01	35.78
Average annual income per household ^d^ (Thousands of €)	29,152.59	27,827.12	27,306.12	−6.33%	4168.03	4327.88	4268.39
**Qualitative Variables**	**2010–2012**						
	**Number**				**Percentage**		
**Exogenous Hospital Variables**							
Hospital Type							
Public	198				86.09%		
Private	20				8.70%		
Public-Private Partnership	12				5.22%		

^a^ Includes diagnostic imaging equipment, radiotherapy and other equipment classified as such. ^b^ Includes the following categories: private individuals, private insurers, civil servant mutual, other public entities, Mutual Insurance Companies for Accidents at Work and Occupational Diseases (MATEP), international agreements and traffic accidents. ^c^ Includes all activities related to the provision and management of health services (primary, specialised and hospital care, public health, clinical research), the provision and management of educational services (infant, primary, secondary, post-secondary and higher education, scholarships, education auxiliary services) and the provision and management of social protection services (those derived from illness and disability, advanced age, survivors, protection of the family, unemployment, housing, social exclusion). ^d^ Corresponding to the year prior to the year in which the study was conducted. Source: Prepared by the authors.

**Table 4:** Technical efficiency of the Spanish National Health System general hospitals, according to ownership. 2010–2012.

	Overall Technical Efficiency			Pure Technical Efficiency			Efficiency of Scale		
	2010	2011	2012	2010	2011	2012	2010	2011	2012
**Public Hospitals** **(*n* = 198)**									
Average	0.739	0.681	0.706	0.831	0.798	0.809	0.894	0.861	0.88
Number of Efficient Hospitals	13	6	8	45	33	32	-	-	-
Percentage of Efficient Hospitals	6.57	3.03	4.04	22.73	16.67	16.16	-	-	-
**Private Hospitals** **(*n* = 20)**									
Average	0.94	0.915	0.941	0.975	0.977	0.977	0.963	0.936	0.961
Number of Efficient Hospitals	11	9	12	17	16	17	-	-	-
Percentage of Efficient Hospitals	55.00	45.00	60.00	85.00	80.00	85.00	-	-	-
**Public-Private Partnership Hospitals** **(*n* = 12)**									
Average	0.928	0.966	0.966	0.937	0.976	0.98	0.99	0.99	0.986
Number of Efficient Hospitals	9	9	7	10	11	11	-	-	-
Percentage of Efficient Hospitals	75.00	75.00	58.33	83.33	91.67	91.67	-	-	-
**Total hospitals (*n* = 230)**									
Average	0.766	0.716	0.740	0.849	0.823	0.833	0.905	0.874	0.892
Number of Efficient Hospitals	33	24	27	72	60	60	-	-	-
Percentage of Efficient Hospitals	14.35	10.43	11.74	31.30	26.09	26.09	-	-	-

Source: Prepared by the authors.

**Table 5:** Relationship between the type of ownership of Spanish National Health System general hospitals and position on the frontier of pure technical efficiency. 2010–2012.

Independent Term	Coefficient	Standard Error	Probability ^c^	*p*-Value
Constant	−1.776	0.323	0.145	<0.001
**Variable ^a^**	**Coefficient**	**Standard Error**	**Odds Ratio**	***p*** **-Value**
Year	−0.165	0.143	0.848	0.250
Equipment per 100 ETC	0.108	0.085	1.114	0.201
Residents per 100 ETC ^b^				0.836
Residents	−0.003	0.011	-	
Residents^2^	0.001	0.000	-	
Percentage of non SNHS discharges	−0.002	0.013	0.998	0.847
Type of hospital				<0.001
Public	Reference	Reference	Reference	
Public Private Partnership	3.732	0.960	41.763	
Private	2.484	0.711	11.989	
FPS Spending (hundreds of €)	−0.149	0.077	0.862	0.053
Private beds per 1000 inhabitants	−0.610	0.853	0.543	0.474
Ageing Index	0.000	0.007	1.000	0.978
Income per Household (Thousands of €)	0.219	0.070	1.245	0.001
**Random Parameters**	**Coefficient**	**Standard Error**		
Second Level				
Independent Term Variance $(\sigma_{u_{0}}^{2}$)	5.660	0.830		
Coefficient Variance per year $(\sigma_{u_{1}}^{2}$)	0.457	0.222		
Covariance between independent term and year $\left(\sigma_{u_{01}}\right)$	−0.326	0.321		
Third Level				
Independent Term Variance $\left(\sigma_{v_{0}}^{2}\right)$	0.386	0.324		
**Extra-Binomial Dispersion**	**Coefficient**	**Standard Error**		
Parameter (θ)	0.389	0.034		

^a^ Year variable is centred on 2010. The other variables are centred on the average. ^b^ Convex quadratic function. ^c^ Probability of being on the frontier of Pure Technical Efficiency of a hospital with an average value in the independent variables in 2010. Source: Prepared by the authors.

## Appendix A

**Table A1:** Ownership and management of Spanish National Health System general hospitals.

Ownership and Type of Management ^a^		Description and Main Characteristics
1. Public Ownership	Without Legal Personality	—Public hospital without legal personality. —Constitutes the major part of the hospital offer of the Spanish National Health System (SNHS). —They are strongly integrated in the Health Services of each region. —Their personnel regime is statutory/official in all cases.
	With Legal Personality	—Public hospital with legal personality. —Contracting according to public or private law, according to the form of management admitted by the Spanish legal system (Consortium, Public Company, Public Health Foundation) adopted by the hospital. —Labour or statutory staff, established case by case.
2. Private Ownership	Private non-profit hospital	—Non-profit private hospital with approval
	Private for profit Hospital	—For profit private hospital with approval
3. Public Private Partnership	Health Administrative Concession (Alzira Model)	—Administrative concession for the construction and management of the building and the provision of health and non-sanitary services for a defined population. —The insurance premium per assigned person includes the payment of the initial investment for the construction of hospitals and health centres, and the provision and renovation of all types of furniture and technological equipment. —The transfer of risks from the public to the private sector is required.
	Private Finance Initiative	—Public law entity with a public works concession for the construction and management of the health building and the provision of non-health services. —Transfer of risks from the public to the private sector is required.

^a^ More information on the Spanish National Health System’s forms of hospital management is available at: Martín, J.J., López del Amo González, M.P., Cabasés Hita, J.M.: La empresa pública en la sanidad. Prestación de sanidad pública por hospitales y ambulatorios privados. Presupuesto y Gasto Público 83, 81–104 (2016). Source: Prepared by the authors.

**Table A2:** Final and alternative models of the analysis of the technical efficiency of Spanish National Health System general hospitals.

Model	Inputs	Outputs	Spearman Correlation Coefficient
Model 1	IB, FP, OHP, NHP	TAD, OC, E, MSP	0.840
Model 2	IB, P&E, (FP + OHP), NHP	TAD, OC, E, MSP	0.994
Model 3	IB, P&E, (FP + OHP + NHP)	TAD, OC, E, MSP	0.959
Model 4	IB, P&E, FP, OHP, NHP	TAD	0.763
Model 5	IB, P&E, FP, OHP, NHP	TAD, OC, E	0.978
Final Model	IB, P&E, FP, OHP. NHP	TAD, OC, E, MSP	

IB: Installed Beds; P&E: Purchases and external services; FP: Faculty Personnel; OHP: Other Healthcare Personnel; NHP: Non Healthcare Personnel; TAD: Total Adjusted Discharges; OC: Outpatient Consultations; E: Non-admitted Emergencies; MSP: Major Surgical Procedures. Source: Prepared by the authors.
